# Malic Enzyme Couples Mitochondria with Aerobic Glycolysis in Osteoblasts

**DOI:** 10.1016/j.celrep.2020.108108

**Published:** 2020-09-08

**Authors:** Wen-Chih Lee, Xing Ji, Itzhak Nissim, Fanxin Long

**Affiliations:** 1 Translational Research Program in Pediatric Orthopedics, The Children’s Hospital of Philadelphia, PA 19104, USA; 2 Division of Genetics and Metabolism, The Children’s Hospital of Philadelphia, Philadelphia, PA 19104, USA; 3 Department of Pediatrics, Biochemistry and Biophysics, University of Pennsylvania, Philadelphia, PA 19104, USA; 4 Department of Orthopaedic Surgery, University of Pennsylvania, Philadelphia, PA 19104, USA; 5 Lead Contact

## Abstract

The metabolic program of osteoblasts, the chief bone-making cells, remains incompletely understood. Here in murine calvarial cells, we establish that osteoblast differentiation under aerobic conditions is coupled with a marked increase in glucose consumption and lactate production but reduced oxygen consumption. As a result, aerobic glycolysis accounts for approximately 80% of the ATP production in mature osteoblasts. *In vivo* tracing with ^13^C-labeled glucose in the mouse shows that glucose in bone is readily metabolized to lactate but not organic acids in the TCA cycle. Glucose tracing in osteoblast cultures reveals that pyruvate is carboxylated to form malate integral to the malate-aspartate shuttle. RNA sequencing (RNA-seq) identifies *Me2*, encoding the mitochondrial NAD-dependent isoform of malic enzyme, as being specifically upregulated during osteoblast differentiation. Knockdown of *Me2* markedly reduces the glycolytic flux and impairs osteoblast proliferation and differentiation. Thus, the mitochondrial malic enzyme functionally couples the mitochondria with aerobic glycolysis in osteoblasts.

## In Brief

Lee et al. discover that mature osteoblasts produce 80% of the energy from glycolysis even under aerobic conditions. *In vivo* metabolic tracing identifies lactate as the predominant fate of glucose in bone. The robust glycolysis is supported by malic enzyme 2 and the malate-aspartate shuttle.

## Graphical Abstract

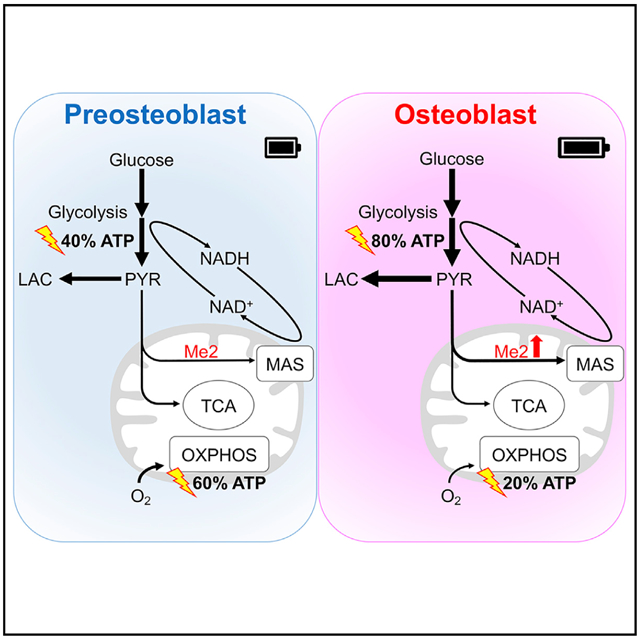

## INTRODUCTION

Proper bone remodeling is essential for maintaining the integrity of bone, and it requires an exquisite balance between bone resorption by osteoclasts and bone formation by osteoblasts. Loss of the balance in favor of bone resorption causes osteoporosis or osteopenia that leads to millions of bone fractures annually (Cummings and Melton, 2002). The current bone anabolic agents teriparatide and abaloparatide, both activating the parathyroid hormone receptor, are effective in increasing bone mineral density and reducing fracture risks, but their use is limited by unresolved concerns about osteosarcoma risks (Cipriani et al., 2012; Ramchand and Seeman, 2018). The newly US Food and Drug Administration (FDA)-approved romosozumab, an antibody against the Wnt inhibitor sclerostin, is dually functional in inducing bone formation and suppressing bone resorption but carries a warning about increased cardiovascular risks (Sølling et al., 2018). Thus, there is a continuing need for developing safe and effective alternative treatments for osteoporosis. Recent studies have increasingly linked diabetes mellitus with increased bone fractures (Schwartz, 2017; Weber et al., 2015). Although the mechanisms underlying bone comorbidity in diabetes are complex, potential disruption of energy metabolism in osteoblasts may lead to impaired osteoblast function (Lee et al., 2017; Napoli et al., 2017). Moreover, the bone anabolic function of PTH and Wnt signaling has been partially attributed to their regulation of osteoblast metabolism (Chen et al., 2019; Esen et al., 2013, 2015; Frey et al., 2015; Karner et al., 2015). These advances have raised the potential that metabolic pathways may be targeted for developing additional bone therapies.

Cellular metabolism in osteoblasts however is just beginning to be understood. Historical studies demonstrated that bone ex-plants as well as freshly isolated calvarial cells from rodents consumed glucose at a brisk rate *in vitro* (Borle et al., 1960; Cohn and Forscher, 1962; Peck et al., 1964). Recent work with radiolabeled glucose analogs has confirmed a significant uptake of glucose by bone in the mouse, further supporting glucose as a main energy substrate for osteoblasts (Wei et al., 2015; Zoch et al., 2016). On the other hand, glutamine and fatty acids were also implicated in osteoblast bioenergetics (Adamek et al., 1987; Biltz et al., 1983). However, the relative contribution of each substrate to energy production in osteoblasts has not been determined. Furthermore, the mechanism for maintaining the NAD+/NADH redox state necessary for sustaining rapid glycolysis in osteoblasts is not known.

Several studies have investigated glucose metabolism in rodent calvarial cells following differentiation *in vitro* but have reported variable results. Whereas one study showed that glucose consumption and lactate production decreased initially but increased later during differentiation, another reported a consistent increase in glycolysis with osteoblast differentiation (Guntur et al., 2014; Komarova et al., 2000). Moreover, studies of osteoblast differentiation from human bone marrow mesenchymal stem cells have produced opposite results. There, osteoblast differentiation was associated with increased oxidative phosphorylation, but with either no change or a decrease in glycolysis (Chen et al., 2008; Shum et al., 2016). Thus, the metabolic changes during osteoblast differentiation remain to be fully established.

In this study, we have refined a culture system for freshly isolated calvarial cells to undergo robust osteoblast differentiation within seven days. We show that aerobic glycolysis is a main bioenergetic mechanism throughout differentiation and especially dominates energy production in mature osteoblasts. We further demonstrate that the malate-aspartate shuttle between mitochondria and the cytosol is necessary for active glycolysis in the osteoblast.

## RESULTS

### Robust Osteoblast Differentiation from Calvarial Cells

**Figure 1.**
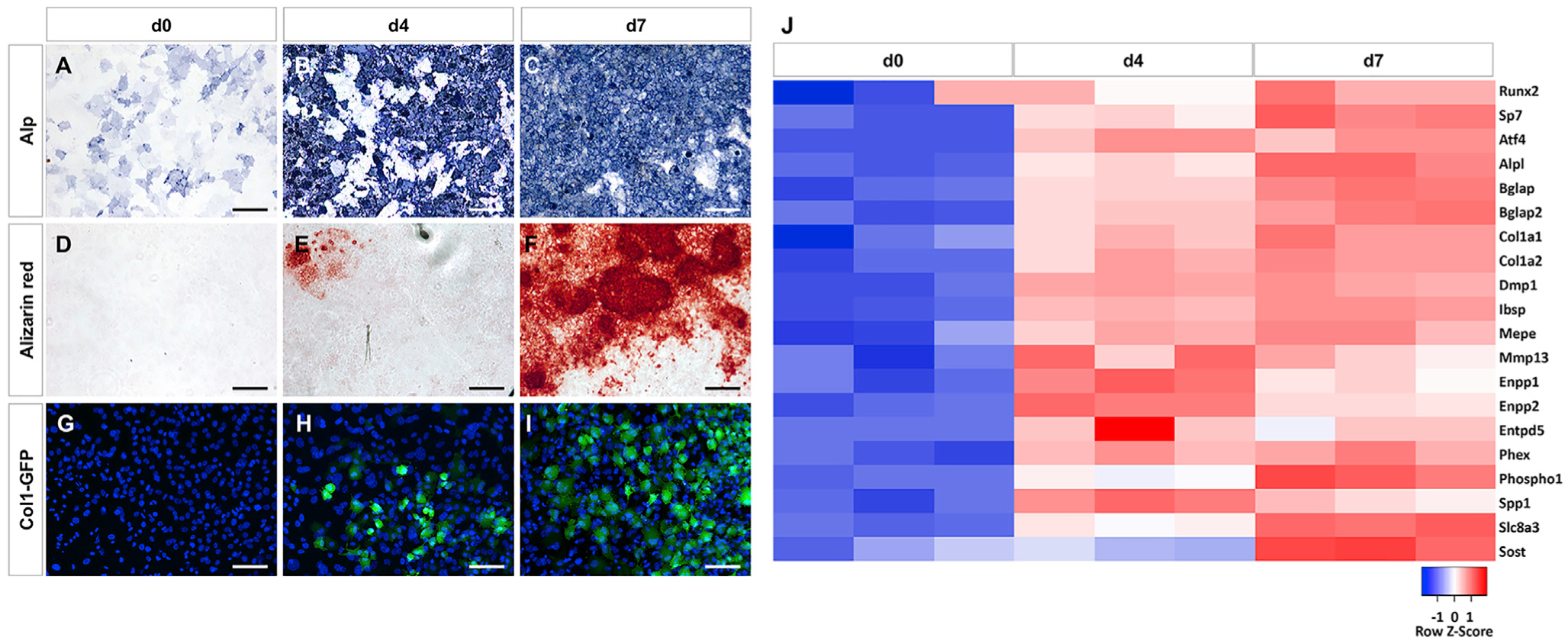
Osteoblast Differentiation from Murine Newborn Calvarial Cells (A–I) Representative views of cell cultures at different days of differentiation with alkaline phosphatase (A–C), Alizarin red staining (D–F), or ColI-GFP expression (G–I). Alp, alkaline phosphatase staining. Scale bar: 50 μm. (J) Heatmap representing relative expression levels of osteoblast marker genes from RNA-seq. Three biological replicates (n = 3) at each time point.

To achieve a reliable and efficient system for osteoblast differentiation, we have optimized both the isolation method of calvarial cells from newborn mice and the culture condition. With the optimized protocol, the calvarial cells before differentiation (day 0) exhibited only faint and scattered staining of alkaline phosphatase (Alp) activity (Figure 1A). After four or seven days of differentiation, however, most cells displayed intense Alp staining (Figures 1B and 1C). Alizarin red staining for minerals detected no signal at day 0, occasional staining at day 4, but wide-spread intensely stained nodules by day 7 (Figures 1D–1F). To confirm osteoblast differentiation, we repeated the procedure with ColI-GFP transgenic mice that express GFP mainly in mature osteoblasts. No GFP was detected at day 0, but an increasing number of GFP+ cells appeared after days 4 and 7 (Figures 1G–1I). Finally, to obtain a global profile of gene expression, we performed RNA sequencing (RNA-seq) experiments with the cells of different stages. Heatmap analyses showed that all known osteoblast markers including Runx2, Sp7, Atf4, Alpl, and Bglap were induced after four days of differentiation, and further upregulated after seven days (Figure 1J). Sost, which is believed to be expressed mainly by osteocytes, was clearly elevated at day 7, indicating that some cells might be transitioning to the more advanced stage. Thus, the current protocol supports robust osteoblast differentiation from calvarial cells within seven days.

### Glucose Is the Major Energy Source for Osteoblasts

**Figure 2.**
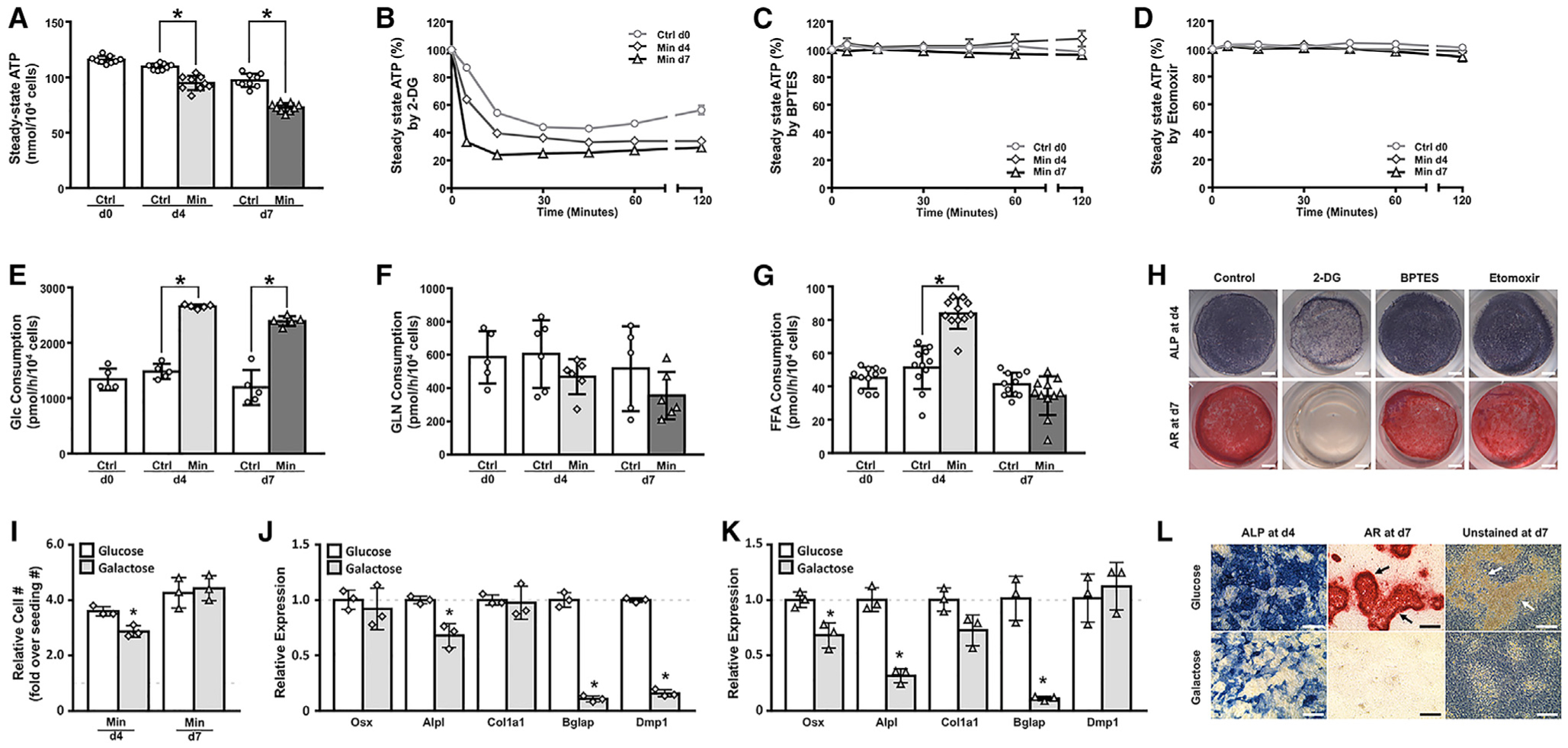
Glucose Dependence of Osteoblast-Lineage Cells Cells were cultured in media supplemented with glucose (Glc), glutamine, and free fatty acids. (A) Steady-state intracellular ATP levels. Ctrl, control media; Min, mineralization media. n = 9. (B–D) Effects of inhibitors on steady-state ATP levels. n = 3. (E–G) Consumption rate of different energy substrates. n ≥ 5. (H) Effects of inhibitors on osteoblast differentiation. Scale bar: 1 mm. (I–K) Effects of galactose or Glc on cell number (I) or osteoblast marker gene expression at day 4 (J) or day 7 (K). Dashed lines denote gene expression levels in Glc. n = 3. (L) Detection of alkaline phosphate activity or mineralized nodules at day 4 or day 7 of differentiation. Note mineralized nodules (arrows) visible with or without AR staining only in Glc-cultured cells. ALP, alkaline phosphatase; AR, alizarin red. Scale bar: 200 μm. *p < 0.05. Error bars: SD.

To explore potential changes in bioenergetics, we first measured the steady-state levels of intracellular ATP (adenosine triphosphate) at different stages of osteoblast differentiation. After being cultured for different days in control or mineralization media, the cells were dissociated and reseeded in fresh media supplemented with glucose, glutamine, and free fatty acids for ATP measurements. The day-4 or −7 differentiated cells showed lower ATP levels than the cells cultured in control media for the same length of time, likely reflecting increased ATP consumption associated with osteoblast differentiation as later analyses showed increased ATP production in the process (Figure 2A; see below). To determine the relative contribution of the major nutrients to bioenergetics, we used 2-DG (2-deoxy-glucose), BPTES, or etomoxir to block the utilization of glucose, glutamine or fatty acids, respectively, and monitored the intracellular ATP levels for up to 2 h. The appropriate dose of each inhibitor was determined by screening a series of dilutions to identify a high concentration that did not cause >5% lethality after 2 h of treatment (see STAR Methods). Inhibition of glucose metabolism by 2-DG abruptly reduced the steady-state ATP level within 30 min in day-0, day-4, and day-7 cells by 56%, 63%, and 75%, respectively (Figure 2B). In contrast, inhibition of glutamine or fatty acid consumption by BPTES or etomoxir, respectively, had no consistent effect on ATP levels for up to 2 h despite slight fluctuations at certain time points that might reflect compensatory reactions (Figures 2C and 2D). Consistent with the ATP measurements, when cultured in media supplemented with all three energy substrates the undifferentiated calvarial cells consumed glucose at approximately three times the rate of glutamine and 30 times that of free fatty acids (Figures 2E–2G). Moreover, glucose consumption was significantly increased after four or seven days of differentiation over the undifferentiated cells, whereas glutamine was not changed and fatty acid consumption was only transiently increased at day 4 of differentiation (Figures 2E–2G). A similar finding that glucose is the main energy substrate for osteoblasts has been reported previously based on a different experimental approach (Wei et al., 2015). Functionally, whereas 2-DG notably suppressed Alp expression at day 4 and abolished mineralization as stained by alizarin red at day 7, BPTES or etomoxir had no obvious effect (Figure 2H). The concentrations of the inhibitors were chosen so that they were sufficiently high but did not cause >10% cell lethality at the end of the culture (see STAR Methods). To test further the functional relevance of increased glycolysis to osteoblast differentiation, we replaced glucose in the media with galactose, which is known to reduce the glycolytic flux as it must be metabolized via the Leloir pathway before entering the glycolytic mainstream (Bustamante and Pedersen, 1977). Consistent with the previous report, galactose was largely sufficient to support cell proliferation as it achieved the same cell number as glucose after seven days of differentiation, and only modestly reduced the number at day 4 (Figure 2I). However, a number of osteoblast-marker genes were suppressed after four or seven days in mineralization media, indicating impairment of osteoblast differentiation (Figures 2J and 2K). Staining for Alp activity at day 4 confirmed a clear deficit in differentiation with galactose; alizarin-red staining at day 7 revealed that galactose blocked the formation of mineralized nodules, which were readily visible even without the staining in cultures with glucose (Figure 2L). These results therefore support that osteoblast lineage cells use glucose as the major energy source, and that the glucose dependence increases with osteoblast differentiation.

### Aerobic Glycolysis Dominates Energy Production in Osteoblasts

**Figure 3.**
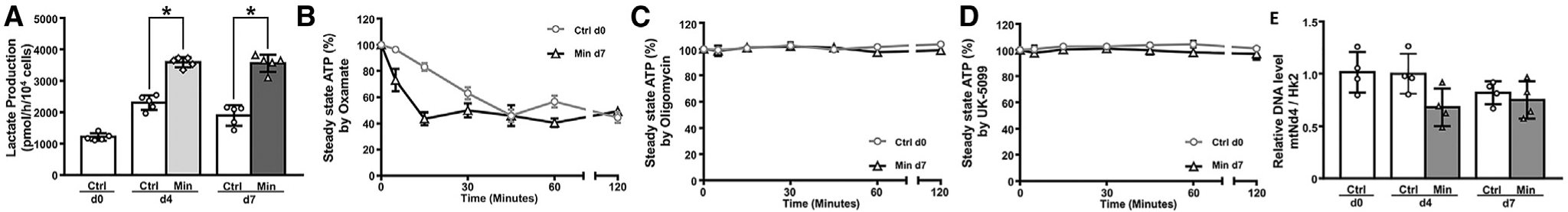
Predominance of Aerobic Glycolysis in ATP Production in Osteoblasts (A) Lactate (Lac) production rate in media supplemented with Glc, glutamine, and fatty acids. n = 3. (B–D) Effects of inhibitors on steady-state ATP levels in media supplemented with Glc and glutamine. n = 3. (E) Relative DNA abundance of mt-ND4 over Hk2 by quantitative PCR (qPCR). n = 4. *p < 0.05. Error bars: SD.

As osteoblasts have been indicated to convert glucose mainly to lactate under aerobic conditions, exhibiting a phenomenon known as aerobic glycolysis, we measured lactate levels in the culture medium. Similar to the increase in glucose consumption, the rate of lactate production per cell after four or seven days of differentiation increased to two-to-three times that of the undifferentiated control cells (Figure 3A). Reliance on aerobic glycolysis predicts glycolysis instead of OXPHOS (oxidative phosphor-ylation) as the main mechanism for energy production in osteoblasts. To test this prediction, we used oxamate, UK-5099, or oligomycin to inhibit lactate dehydrogenase, mitochondrial pyruvate carrier, or ATP synthase, respectively. Inhibition of pyruvate-to-lactate conversion by oxamate reduced intracellular ATP levels by ~30% in the day-0 cells and ~60% in the day-7 osteoblasts (Figure 3B). In contrast, inhibition of pyruvate entry to the mitochondria by UK-5099, or inhibition of mitochondrial ATP production by oligomycin, did not have any effect on the steady-state ATP levels (Figures 3C and 3D). Measurements of the relative copy number of mt-Nd4, a representative mitochondrial gene, over the nuclear gene Hk2 showed that the abundance of mitochondria per cell stayed relatively constant during differentiation (Figure 3E). Thus, aerobic glycolysis is mainly responsible for energy production in osteoblasts.

**Figure 4.**
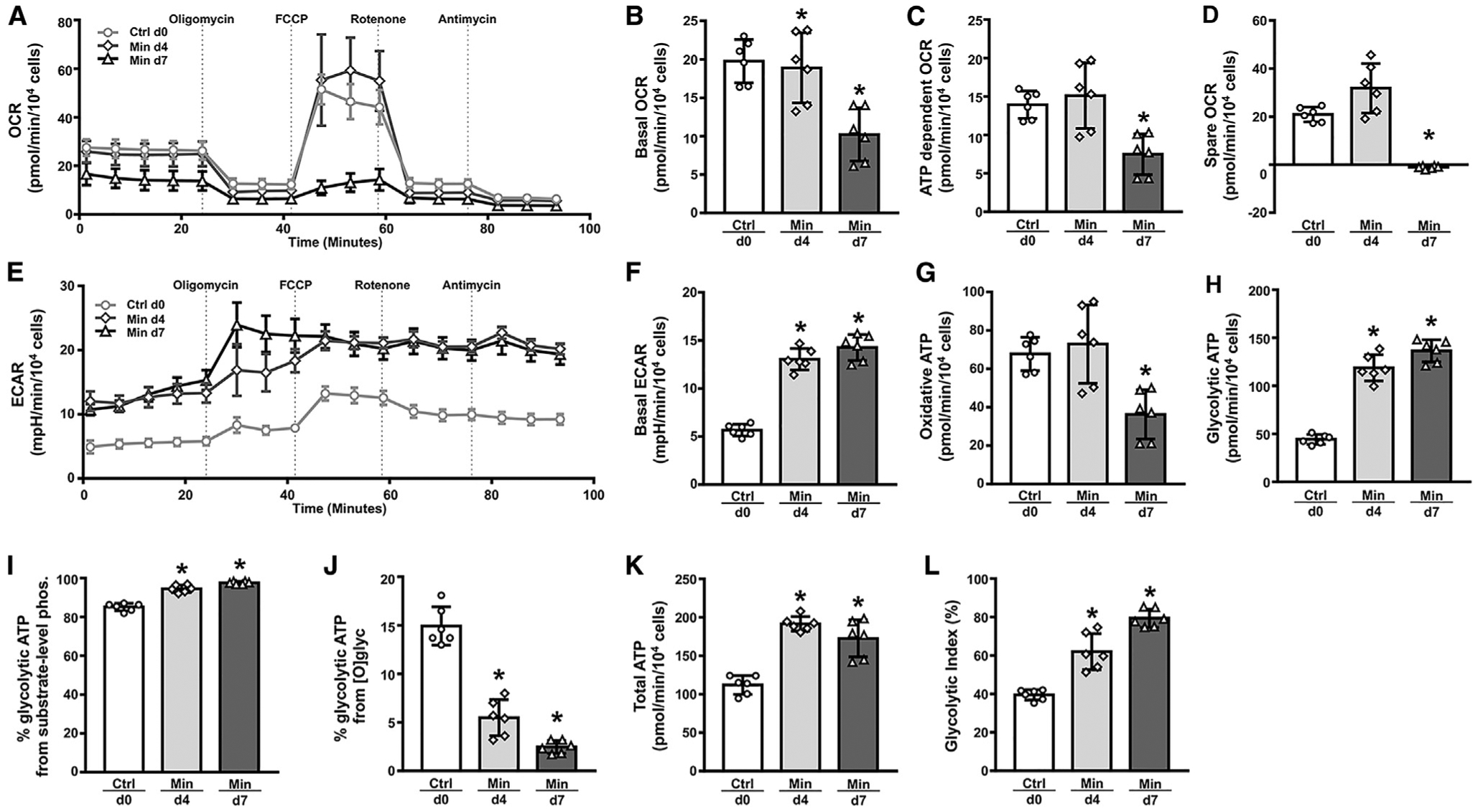
Metabolic Changes during Osteoblast Differentiation (A) A representative graph for Seahorse measurements of oxygen consumption rate (OCR). (B–D) Quantification of OCR parameters. (E) A representative graph of extracellular acidification rate (ECAR). (F) Quantification of basal ECAR. (G and H) Theoretical ATP production from OXPHOS (G) or glycolysis (H). (I and J) Relative contribution to glycolytic ATP by substrate-level phosphorylation (I) or oxidation of NADH transferred from cytoplasm (J). (K) Total ATP production from glycolysis and OXPHOS. (L) Relative contribution of glycolysis to total ATP production. n = 6. *p < 0.05. Error bars: SD.

To gain further insights into the metabolic changes during osteoblast differentiation, we performed Seahorse analyses. As we detected no difference in their metabolic behavior among day-0, day-4, or day-7 control cells, we include here only the day-0 control cells for comparison with the differentiated cells. The basal oxygen consumption rate (OCR) was similar between day-0 and day-4 cells, but significantly reduced in the day-7 osteoblasts (Figures 4A and 4B). Moreover, ATP production OCR and spare capacity OCR were both decreased in the day-7 cells (Figures 4C and 4D). In contrast to OCR, the extracellular acidification rate (ECAR) was significantly higher in day-4 and day-7 than day-0 cells (Figures 4E and 4F). The increased ECAR was consistent to the greater lactate production rate associated with osteoblast differentiation as shown above. Together, the data indicate that osteoblast differentiation is associated with increased glycolysis but reduced oxidative phosphorylation.

We next calculated the theoretical ATP production from either glycolysis or OXPHOS based on ECAR or OCR from Seahorse analyses. Such calculations showed that ATP from OXPHOS decreased by ~50% in day-7 versus day-0 cells, whereas ATP from glycolysis increased by ~150% in day-4 or day-7 cells compared to day-0 cells (Figures 4G and 4H). Further breakdown of the glycolytic ATP production indicated that a predominant majority resulted from substrate-level phosphorylation in the cytoplasm, ranging from 85% in day-0 to 95% in day-4 to 98% in day-7 cells, whereas the contribution from mitochondrial oxidation of NADH transported from the cytoplasm progressively decreased with differentiation (Figures 4I and 4J). The increase in glycolytic ATP production resulted in a significant increase in total ATP output in the day-4 and day-7 cells (Figure 4K). Furthermore, the contribution of glycolysis toward total ATP production increases from 40% in day-0 cells to 60% or 80% in day-4 or day-7 cells, respectively (Figure 4L). These results therefore demonstrate that glycolysis supplies most of the energy in mature osteoblasts.

### Glucose Is Mainly Converted to Lactate and Contributes to the Malate-Aspartate Shuttle in Osteoblasts

**Figure 5.**
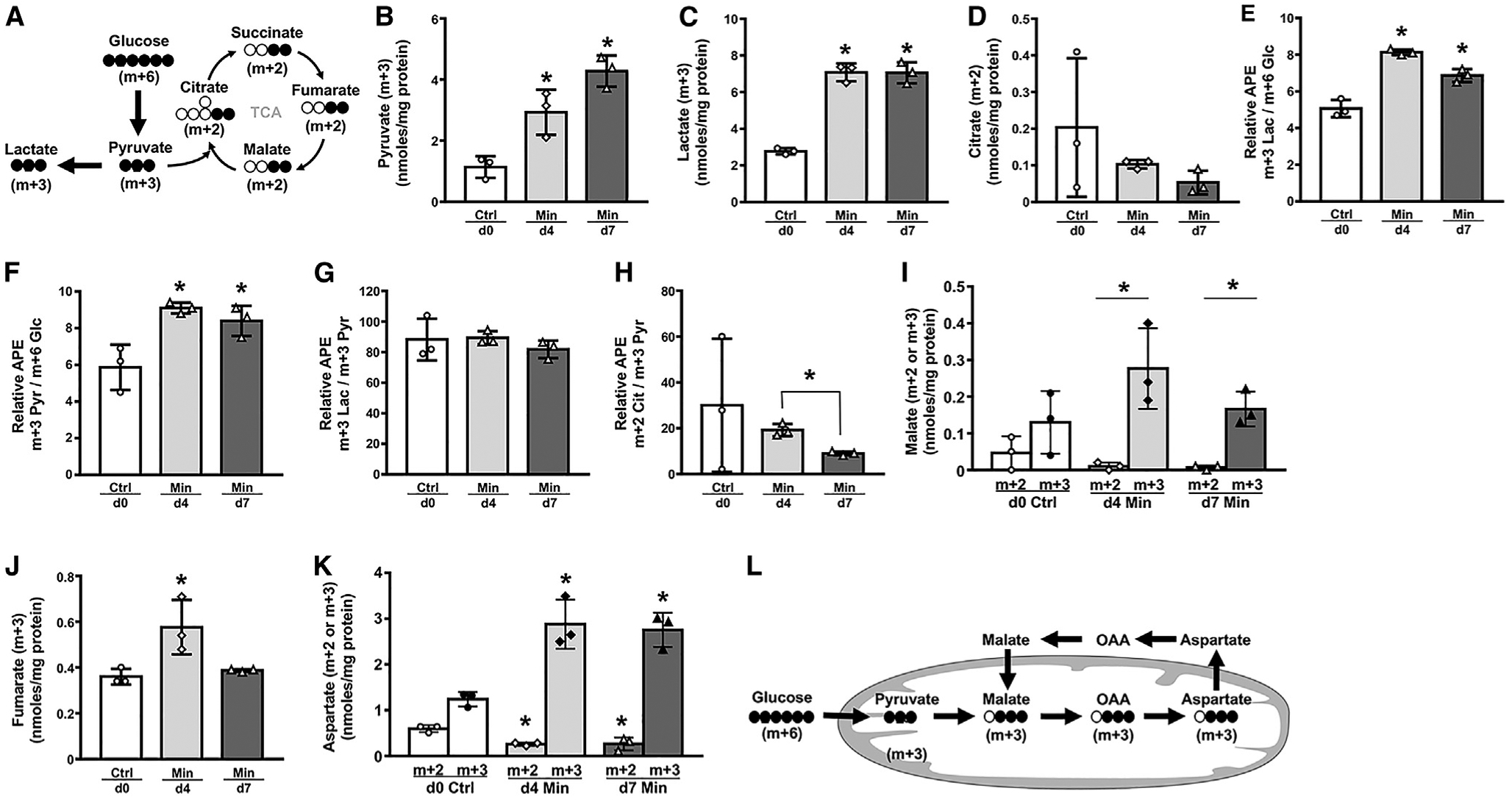
Metabolic Tracing of ^13^C-Glc in Cell Cultures (A) A diagram for carbon tracing through glycolysis or TCA cycle. Closed circles: ^13^C; open circle: ^12^C. (B–D and I–K) Abundance of ^13^C-labeled metabolites normalized to cellular protein. (E–H) Relative enrichment ratio of key metabolites over precursor. APE, atom percent excess. (L) A diagram for carbon tracing through the malate-aspartate shuttle. n = 3. *p < 0.05, versus day 0 unless otherwise indicated. Error bars: SD.

To directly examine the metabolic fate of glucose, we performed isotope-tracing experiments with uniformly labeled D-glucose (^13^C_6_-Glc) and analyzed intracellular metabolites by mass spectrometry. The fully labeled glucose (m+6) was expected to produce fully labeled pyruvate (m+3) through glycolysis, which could then convert to lactate (m+3) in the cytosol or produce citrate (m+2) in mitochondria to fuel TCA (tricarboxylic acid) cycle metabolism (Figure 5A). After 30 min of incubation with ^13^C_6_-Glc, the amount of intracellular pyruvate (m+3) was significantly higher in day-4 than day-0 cells and was further increased in the day-7 osteoblasts (Figure 5B). Moreover, the intracellular lactate (m+3) amount increased by 150% in day-4 and day-7 over day-0 cells (Figure 5C). In contrast, the amount of citrate (m+2) was less than 10% of lactate (m+3) in day-0 cells and further decreased with differentiation (Figure 5D). Succinate (m+2) or fumarate (m+2), which are TCA metabolites downstream of citrate (m+2), were not detectable at any stage. We next calculated the relative conversion rate of key metabolites by normalizing their labeling percentage to that of the precursor. Such calculations showed that glucose was converted to lactate at a significantly higher rate in day-4 and day-7 than day-0 cells (Figure 5E). Interestingly, the increased rate was driven by the greater production of pyruvate from glucose as the conversion from pyruvate to lactate stayed constant throughout differentiation (Figures 5F and 5G). Finally, the generation of citrate from pyruvate by pyruvate dehydrogenase was significantly reduced in day-7 versus day-4 cells (Figure 5H). The results therefore provide direct evidence that glycolysis is accelerated to produce lactate in osteoblasts with little contribution to the TCA cycle via pyruvate dehydrogenase.

Analyses of the tracing data revealed additional fates for glucose. Malate (m+2) was barely detectable in day-0 cells and further deceased with differentiation, again confirming minimal contribution of glucose to the TCA cycle through pyruvate dehydrogenase. Interestingly however, malate (m+3) was present at a significantly higher level than malate (m+2) in day-4 and day-7 cells (Figure 5I). Fumarate (m+3), which interconverts with malate (m+3), was also readily detected in all cells (Figure 5J). Furthermore, aspartate (m+3), which can be derived from malate (m+3), was readily detectable in day-0 cells and more than doubled in day-4 and day-7 cells (Figure 5K). These results indicate that glucose-derived pyruvate is converted to malate through carboxylation, perhaps to engage the malate-aspartate shuttle in osteoblasts (Figure 5L).

### Glycolytic Genes Are Coordinately Upregulated during Osteoblast Differentiation

**Figure 6.**
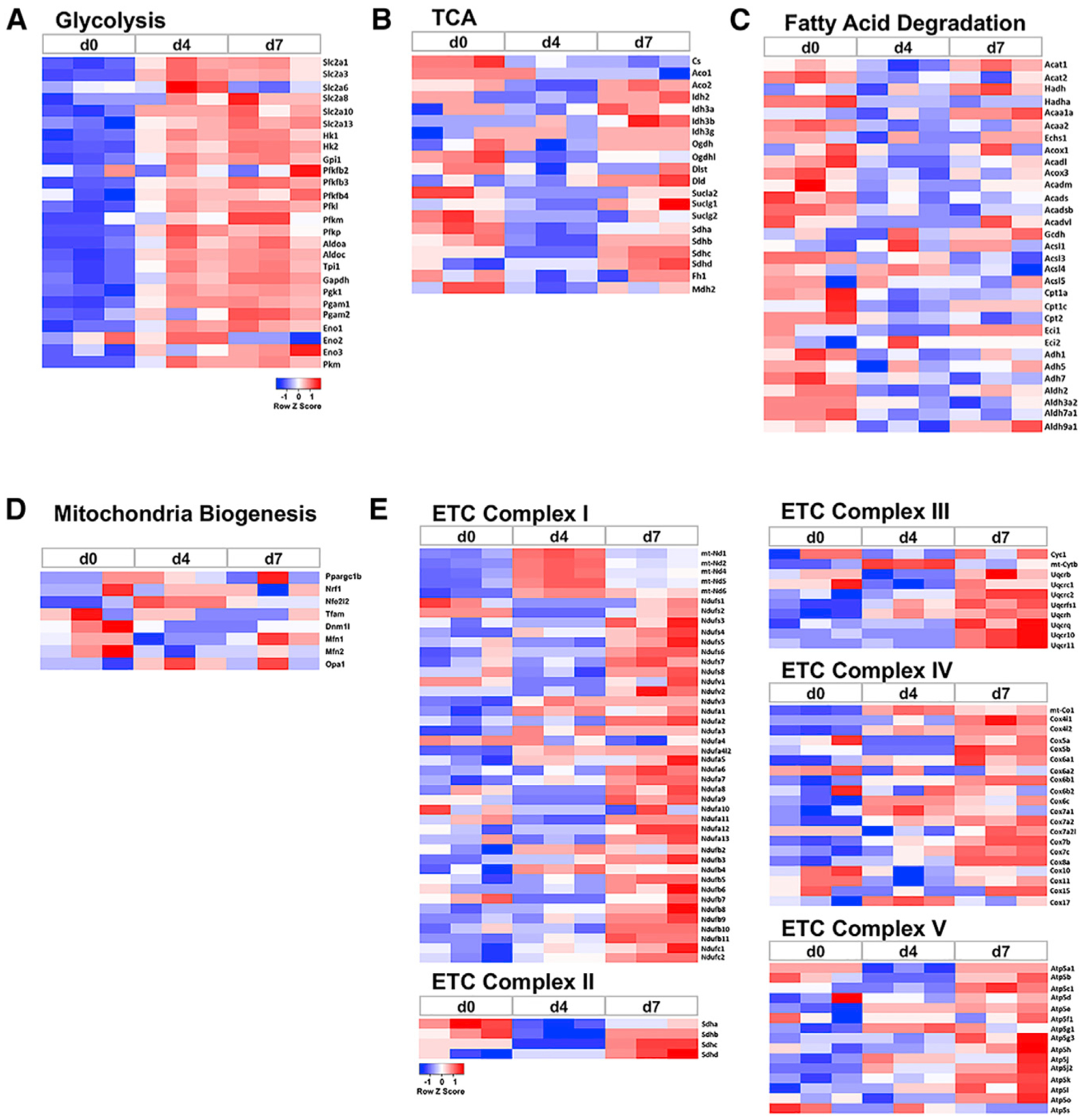
Differential Expression of MetabolicGenes during Osteoblast Differentiation Heatmaps were derived fromRNA-seq experiments with three biological replicates at each stage. (A) Glycolytic genes. (B) TCA cycle genes. (C) Fatty acid catabolism genes. (D) Mitochondria biogenesis genes. (E) Electron transport chain (ETC) genes.

To gain insight into the molecular basis for metabolic reprogramming during osteoblast differentiation, we analyzed the transcriptomic changes obtained by RNA-seq. A cutoff of RPKM (reads per kilobase of transcript, per million mapped reads) >2 and fold changes >2 between any two of the three stages resulted in 1,214 genes. Analyses of those genes with KEGG Mapper identified metabolic pathways (ko01100) as the top category encompassing ~10% of all changes during osteoblast differentiation. Specifically, essentially all glycolysis genes were upregulated after four days of differentiation and further enhanced at day 7 (Figure 6A). In contrast, the TCA cycle genes as well as the fatty acid catabolism genes were largely suppressed at day-4 compared to day-0, although some of the genes rebounded to the day-0 level at day 7 (Figures 6B and 6C). In keeping with the relatively stable load of mitochondria in the cells, the genes regulating mitochondrial biogenesis did not exhibit consistent changes during differentiation (note that Ppargc1a was virtually undetectable at all stages and thus not included in the heatmap; Figure 6D). Unexpectedly, however, most genes encoding the subunits of the mitochondrial electron transport chain (ETC) complexes I to V were significantly upregulated at day 7 compared to day 0, likely reflecting a compensatory response to the suppressed OXPHOS in day-7 cells (Figure 6E). Finally, the genes for fatty acid synthesis or amino acid metabolism, as a whole, did not exhibit an obvious change pattern during differentiation (Figures S1A and S1B). The gene expression changes therefore support the increased glycolysis associated with osteoblast differentiation.

### Me2 Is Necessary for Glycolysis in Osteoblasts

**Figure 7.**
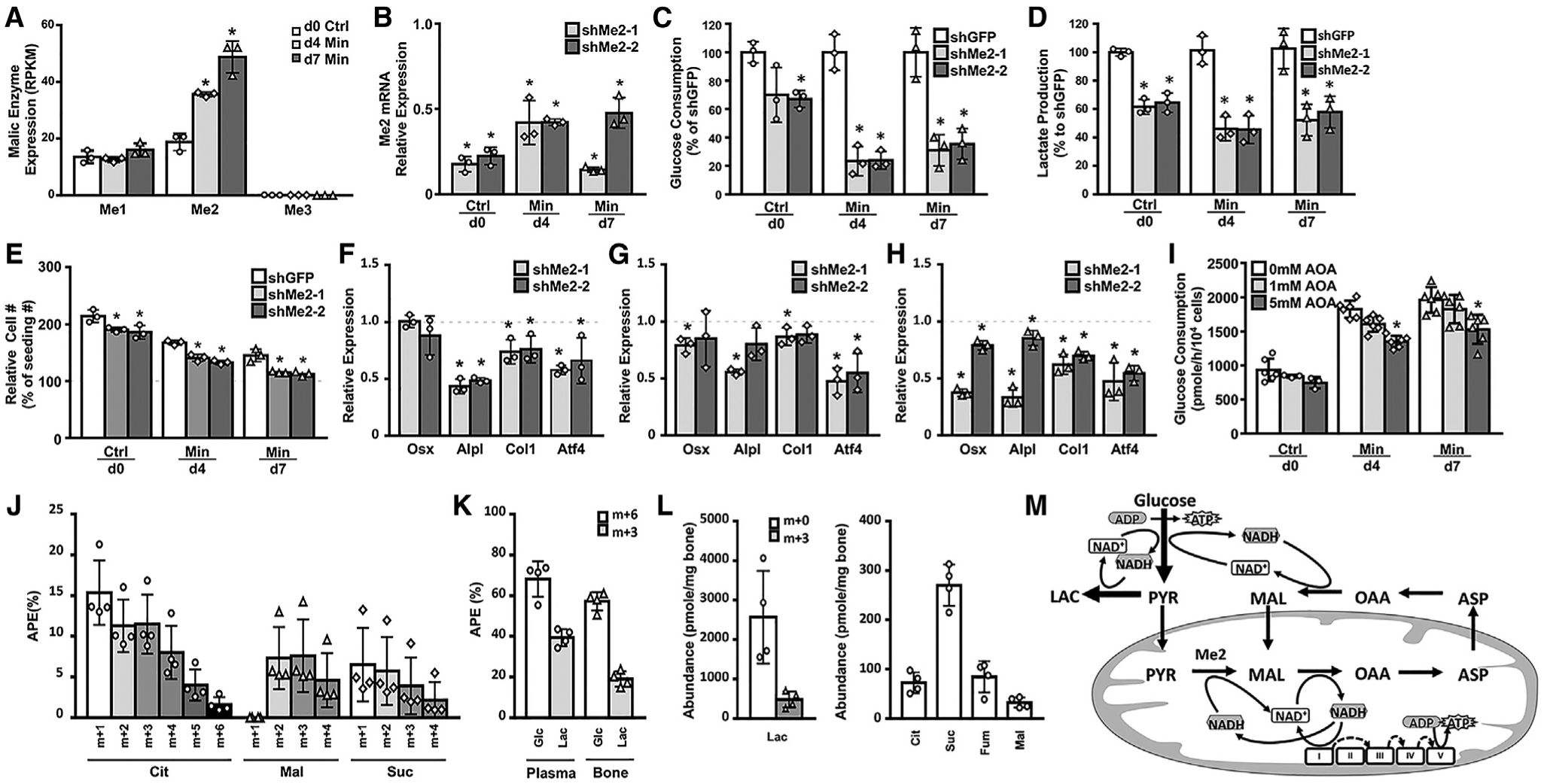
Requirement of Me2 for Aerobic Glycolysis in Osteoblasts (A) Expression levels of malic enzyme genes determined by RNA-seq. *Significant difference from day 0. (B) Knockdown efficiency of shRNA for Me2. shRNA for GFP as control. *Significant difference from shGFP control. (C–H) *Significant difference from shGFP control. (C and D) Effects of Me2 knockdown on Glc consumption (C) and Lac production (D). (E) Effects of Me2 knockdown on cell numbers. (F–H) Effects of Me2 knockdown on gene expression relative to control infected with shGFP control virus in day-0 (F), day-4 (G), or day-7 (H) cells. Dashed lines indicate levels in shGFP-infected cells. (A–H) n = 3. (I) Effects of AOA on Glc consumption. *Significant difference from 0 mM. n ≥ 3. (J) Relative enrichment of TCA cycle organic acids in the plasma of mice as determined by mass spectrometry at 60 min after ^13^C_6_-Glc injection through the tail vein. APE, atom percent excess; Cit, citrate; Mal, malate; Suc, succinate. (K) Relative enrichment of Glc and Lac in plasma or bone as determined by mass spectrometry. (L) Absolute abundance of organic acids in bone as determined by mass spectrometry. Note that only unlabeled TCA cycle organic acids were detected. (J–L) n = 4. (M) A model for aerobic glycolysis driven by the malate-aspartate shuttle in osteoblasts. *p < 0.05. Error bars: SD.

RNA-seq further revealed that *Me2*, encoding an NAD+-dependent mitochondrial isoform of malic enzyme, was expressed at the highest level among the three family members and further induced with osteoblast differentiation (Figure 7A). In contrast, Me1, encoding the NADP+-dependent cytosolic malic enzyme, did not change, whereas Me3 for the NADP+-dependent mitochondrial form was hardly expressed (RPKM < 0.2 in all samples; Figure 7A). The cytosolic or mitochondrial malic enzymes convert pyruvate to malate in a reversible manner. As we have detected a notable contribution of glucose to the malate-aspar-tate shuttle (which is known to regulate the cytosolic NAD+/NADH redox state), we tested a potential role for Me2 in modulating the glycolytic flux in the osteoblast lineage. Knock down of Me2 with two independent shRNA constructs reduced its mRNA level by >50% at all three differentiation stages (Figure 7B). As a result, glucose consumption per cell was consistently reduced by ~30% in day-0 cells, and by ~70% in day-4 and day-7 differentiated cells (Figure 7C). Likewise, Me2 knockdown decreased lactate production per cell by 40% to 50% at all three stages (Figure 7D). During the 24-h culture for glucose and lactate measurements, we noted that the Me2-deficient cells propagated less than the control cells, indicating that Me2 knockdown likely impaired cell proliferation (Figure 7E). In addition, the knockdown also impaired osteoblast differentiation, as both constructs reduced the mRNA level of Atf4 at all three stages, Alpl, Col1a1 in day-0 and day-7 cells, along with Osx (official gene name Sp7) in day-7 cells (Figures 7F, 7G, and 7H). Knock down of Me1, on the other hand, did not impair glucose consumption or lactate production, thus highlighting the specific function of Me2 in promoting glycolysis (Figure S2). Finally, inhibition of the malate-aspartate shuttle with aminooxyacetate (AOA), which targets aspartate aminotransferase, dose dependently reduced glucose consumption in day-4 and day-7 cells (Figure 7I). The data therefore provide evidence that funneling glucose carbons to the malate-aspartate shuttle via Me2 is important for boosting glycolysis in osteoblasts.

### Glucose Contributes Minimally to the TCA Cycle in Murine Cortical Bone

The data so far establishes aerobic glycolysis as a prominent metabolic feature of osteoblasts *in vitro*. To determine the physiological relevance of the observation, we performed glucose-tracing experiments in the mouse. Briefly, mice were injected with ^13^C_6_-Glc through the tail vein at 60 min before sacrifice for the plasma and the femoral cortical bone to be extracted for metabolites. The cortical bone was chosen because it contains mostly osteoblasts and osteocytes with few other cell types. Quantitative analyses of the plasma by mass spectrometry detected clear enrichment of the ^13^C-labeled citrate, malate, and succinate, as well as ~65% enrichment of glucose (m+6) and ~40% lactate (m+3), indicating active glycolysis and TCA cycle metabolism of ^13^C_6_-Glc in the body (Figures 7J and 7K). However, in the bones of the same animals, despite ~60% enrichment of glucose (m+6) and ~20% lactate (m+3), no ^13^C-labeling of citrate, succinate, fumarate, or malate was detected even though those organic acids themselves were readily detectable (Figures 7K and 7L). Thus, glucose is predominantly metabolized to lactate with little contribution to the TCA cycle in the murine cortical bone. As a whole, the data support a model wherein aerobic glycolysis is the predominant bioenergetic pathway in osteoblasts in which Me2 plays a critical role by fueling an active malate-aspartate shuttle (Figure 7M).

## DISCUSSION

We have conducted a comprehensive analysis of the metabolic profile in osteoblasts. The data indicate that glucose instead glutamine or fatty acids is the principal energy source for osteoblasts in culture. Metabolic tracing with labeled glucose shows that glucose is predominantly metabolized to lactate with contribution to the TCA cycle in osteoblasts *in vitro* and cortical bone *in vivo*. The metabolic features of osteoblasts are coupled with wholesale upregulation of the glycolytic genes during osteoblast differentiation. Furthermore, genetic knockdown of mitochondrial malic enzyme indicates that the malate-aspartate shuttle is necessary to sustain the glycolysis flux in osteoblasts. Thus, the present study sheds light on the metabolic wiring osteoblasts.

The study also provides insights into fatty acid utilization osteoblasts. Consistent with previous reports, we have detected fatty acid uptake by calvarial cells before and after osteoblast ferentiation (Frey et al., 2015). However, the uptake amount approximately 30-fold less than that of glucose even with palmitate and oleic acid supplemented at physiological levels. More importantly, inhibition of mitochondrial fatty acid oxidation either etomoxir or oligomycin had little or no effect on steady-state ATP levels. Although we cannot rule out that compensation from glycolysis might mask the mitochondrial energy contribu tion, the data nonetheless indicate that such contribution is dispensable for energy homeostasis. Interestingly, fatty acid uptake peaked at day 4 of differentiation but declined by day 7 to the base level as seen in the undifferentiated cells. A transient increase in fatty acid oxidation at day 4 may explain the ~10% suppression of steady-state ATP levels by oligomycin specifically observed at that stage. Thus, fatty acids oxidation may contribute to osteoblast bioenergetics in a stage-specific manner, but generally appears to play an auxiliary role.

In addition, the current work clarifies the potential contribution of glutamine to energy production in osteoblasts. Glutamine uptake was detected in calvarial cells but did not increase with osteoblast differentiation, indicating that glutamine normally may not play a major role in meeting the increased energy demand in mature osteoblast. In keeping with this view, inhibition of glutamine catabolism with BPTES had no effect on the steady-state ATP levels. The notion is also consistent with our previous finding that inhibition of glutamine catabolism does not affect basal bone mass in the mouse even though it suppresses the excessive bone formation caused by hyperactive Wnt signaling (Karner et al., 2015). A more recent study linked increased glutamine uptake with specification of skeletal stem cells toward the osteoblast lineage but did not report a specific role in energy production (Yu et al., 2019). Collectively, the data to date indicate that glutamine has a limited role as a direct energy substrate in osteoblasts under normal conditions.

The comprehensive metabolic profiling was enabled by our optimized protocol for osteoblast differentiation. Multiple assays including visual inspection of nodule formation, mineral staining, and RNA-seq indicate that the calvarial cells reliably undergo robust osteoblast differentiation within seven days of culture in the current study. In contrast, previous protocols required a minimum of 14 days of induction to achieve differentiation (Guntur et al., 2014; Komarova et al., 2000). Another key difference between the studies lies in how the metabolic data were acquired and normalized. Previously, the metabolic measurements were made directly in the culture wells following differentiation and then normalized to either cell number or protein content recovered from the wells (Guntur et al., 2014; Komarova et al., 2000). However, in our experience, mineralization makes it difficult to fully recover the cells or cellular proteins from the cultures. Therefore, we have chosen to perform all metabolic studies after reseeding of the cells dissociated from the cultures, and to normalize the data to the reseeded cell number. The technical differences could account for the discrepant findings, particularly regarding oxygen consumption, which was previously shown to increase with osteoblast differentiation (Guntur et al., 2014; Komarova et al., 2000).

The reliance of glycolysis for energy production in osteoblasts is counterintuitive as less ATP is produced from each glucose molecule through glycolysis than the TCA cycle. Glycolysis produces a net gain of 2 ATP per glucose molecule through substrate-level phosphorylation, but additional ATP may be generated through oxidative phosphorylation after NADH derived through glycolysis is translocated to the mitochondria via the malate-aspartate shuttle. Our calculations from the Seahorse data indicate that glycolysis produces approximately 80% of the energy in mature osteoblasts. More over, 98% of the glycolytic ATP production in those cells results from substrate-level phosphorylation in the core glycolysis pathway (Table S1; [PPR_glyc_ * ATP/lactate] in equation for glycolytic ATP). The marked increase in glycolysis leads to an overall increase in ATP production even though OXPHOS is diminished in mature osteoblasts. These results therefore resolve the apparent energy paradox about aerobic glycolysis in osteoblasts.

A main finding from the Seahorse assays is that mature osteoblasts exhibit little spare respiration capacity in response to the mitochondrial uncoupling reagent FCCP (carbonyl cyanide-p-trifluoromethox- yphenyl-hydrazon). The failure to increase oxidative phosphorylation could indicate either the ETC capacity or the reducing equivalent (NADH or FADH_2_) from the TCA cycle is limiting. As RNA-seq showed that virtually all genes encoding the ETC subunits were upregulated in mature osteoblasts, we consider it unlikely that the ETC capacity became limiting after differentiation. On the other hand, Pdk1, which encodes pyruvate dehydrogenase kinase suppressing the activity of pyruvate dehydrogenase, was expressed at four times higher in osteoblasts than preosteoblasts. Moreover, intracellular pyruvate accumulated at a higher level with the progression of osteoblast differentiation. Thus, suppression of pyruvate dehydrogenase activity may restrict pyruvate from entering the TCA cycle, resulting in less NADH or FADH2 to fuel oxidative phosphorylation.

The present study provides insight about the mechanism for maintaining the highly glycolytic state in osteoblasts. The critical role of Me2 may be explained by funneling pyruvate into the malate-aspartate shuttle that reoxidizes the cytoplasmic NADH to NAD+. However, as Me2 knockdown reduced glycolysis more severely than the shuttle inhibitor AOA did, we cannot rule out that Me2 may perform additional activities to support glycolysis. We did not detect direct conversion of pyruvate to malate in bone through carbon tracing, but this could be due to insufficient enrichment of ^13^C-glucose *in vivo*. Although the phosphate glycerol shuttle is also known to regenerate NAD+ from NADH in certain cells, we consider this unlikely here as the key enzyme Gpd1 is barely detectable in the osteoblast lineage cells (RPKM < 0.3 at all stages). It is worth noting that oxamate, though commonly used as an inhibitor of lactate dehydrogenase, also inhibits aspartate aminotransferase (Thornburg et al., 2008). Thus, the strong suppression of oxamate on intracellular ATP levels in osteoblasts may result from simultaneous inhibition of both lactate dehydrogenase and the malate-aspartate shuttle. Future studies are warranted to determine the relative contribution of each mechanism to cytoplasmic NADH reoxidation in osteoblasts. Overall, the study identifies aerobic glycolysis as the principal bioenergetic pathway in normal osteoblasts, and thus provides a foundation for future investigations into bone metabolism in pathological conditions such as diabetes. Further elucidation of the relationship between glycolysis and the malate-aspartate shuttle may uncover molecular targets for developing additional bone-enhancing therapies.

## STAR★METHODS

### RESOURCE AVAILABILITY

#### Lead Contact

Further information and requests for resources and reagents should be directed to and will be fulfilled by the Lead Contact, Fanxin Long (longf1@email.chop.edu).

#### Materials Availability

This study did not generate new unique reagents.

#### Data and Code Availability

The RNA-seq data generated during this study are available at Gene Expression Omnibus (GEO: GSE154991, https://www.ncbi.nlm.nih.gov/geo/query/acc.cgi?acc=GSE154991)

### EXPERIMENTAL MODEL AND SUBJECT DETAILS

All primary calvarial cells were isolated from newborn pups of the C57BL/6J (wild type or ColI-GFP) mouse strain at 1–5 days of age; both male and female pups were used. Use of the animals were approved by the Animal Studies Committee at Washington University in St. Louis School of Medicine and the IACUC Committee at The Children’s Hospital of Philadelphia.

### METHOD DETAILS

#### Cell isolation, culture and osteoblast differentiation

Isolation of calvarial preosteoblasts was modified from a previous protocol (Jonason and O’Keefe, 2014). Briefly, calvaria were dissected free of periosteum from neonatal (P1-P5) C57BL/6J wild type or ColI-GFP (Col1a1*2.3-GFP) mice (Kalajzic et al., 2002). Each calvarium was sequentially digested with 0.6 mL of 4 mg/ml collagenase I (Sigma, C0130) dissolved in PBS for multiple rounds of 15 mins at 37°C with gentle shaking at 100 rpm. Cells were collected from the second through fourth digestion and filtered with 70 μm strainer before being centrifuged and seeded at 4×10^4^ cells/cm^2^ in ascorbic acid-free MEMα (Thermo, A10490) supplemented with 10% FBS (Thermo, 26140087) and Penicillin-Streptomycin (Thermo, 15140122). The cells can be passaged once in the same culture condition to increase the cell number. After reaching 100% confluency usually after 3 days, the cells were switched to MEMα containing 10 or 4 mM β-glycerol phosphate (Sigma, G9422) and 50 ug/ml ascorbic acid (Sigma, A4544) with daily changes of media for osteoblast differentiation. Cells after 4 or 7 days of differentiation in 10-cm culture dishes were dissociated first with 4 mg/ml collagenase I in PBS for 30 or 45 minutes, respectively, and then with 0.25% trypsin for 10–15 mins. The cells were then collected and filtered with 70 μm strainer before being reseeded for subsequent studies. In certain differentiation assays, 100 μM 2-DG, or 10 μM BPTES, or 200 μM Etomoxir, or 5.5 mM galactose in place of glucose was added to the mineralization medium.

#### High throughput RNA-sequencing

Total RNA was isolated with QIAGEN RNeasy Kit. Library construction, high-throughput sequencing and bioinformatics were performed by Genome Technology Access Center at Washington University School of Medicine. RNA-seq reads were aligned to the Ensembl release 76 top-level assembly with STAR version 2.0.4b. Gene counts were derived from the number of uniquely aligned unambiguous reads by Subread:featureCount version 1.4.5. Transcript counts were produced by Sailfish version 0.6.3. Sequencing performance was assessed for total number of aligned reads, total number of uniquely aligned reads, genes and transcripts detected, ribosomal fraction known junction saturation and read distribution over known gene models with RSeQC version 2.3. All gene-level and transcript counts were then imported into the R/Bioconductor package EdgeR and TMM normalization size factors were calculated to adjust for samples for differences in library size. Genes or transcripts not expressed in any sample were excluded from further analysis. The TMM size factors and the matrix of counts were then imported into R/Bioconductor package Limma and weighted likelihoods based on the observed mean-variance relationship of every gene/transcript and sample were then calculated for all samples with the voomWithQualityWeights function. Performance of the samples was assessed with a spearman correlation matrix and multi-dimensional scaling plots. Gene/transcript performance was assessed with plots of residual standard deviation of every gene to their average log-count with a robustly fitted trend line of the residuals. Generalized linear models were then created to test for gene/transcript level differential expression. Differentially expressed genes and transcripts were then filtered for FDR adjusted p values less than or equal to 0.05. Heatmaps were generated by Heatmapper (http://www.heatmapper.ca/).

#### ATP, glucose and lactate measurements

Custom media were used for all metabolic studies. A medium free of glucose, glutamine, pyruvate, phenol red, and sodium bicarbonate was based on MEMα with no nucleosides (Thermo, 10490) and custom produced (GIBCO). The medium was first reconstituted with sodium bicarbonate with pH adjusted to 7.4. Complete MEMα (cMEMα) medium was then prepared by adding fresh ingredients to achieve 5.5 mM glucose, 2 mM glutamine, 1 mM pyruvate and 10% FBS. For certain experiments, cMEMα medium was supplemented with 10 mM FFA: 3 mM BSA to a final concentration of 100 μM each of palmitate and oleate.

For intracellular ATP measurements, cells were seeded at 1×10^5^ cells/cm^2^ in 96-well plate for 4 hours before switching to 100 μl of fresh cMEMα for one hour. The different compounds were then added for 5–120 mins to assess the effect on intracellular ATP levels as measured by CellTiter-Glo® Luminescent Cell Viability Assay (Promega, G7570) and normalized to the number of cells seeded. Cell lethality was detected by staining with 4 μM propidium iodide (Thermo, P1304MP) in HBSS (Thermo, 14025) for 1 hour after each drug treatment. Drug concentrations were selected to ensure < 5% lethality after 2 hr of treatment: 2-DG, 100 mM; BPTES, 20 μM; Etomoxir, 200 μM; Oligomycin: 2 μM; Oxamate: 100 mM; UK-5099: 100 μM.

For glucose, glutamine, free fatty acid and lactate measurements, cells were seeded at 1×10^5^ cells/cm^2^ in 6-well plate for 4 hours before being rinsed once with cMEMα medium with 100 μM each of palmitate and oleate and then incubated in 2 mL of the same medium per well for 24 hours. Glucose, glutamine, free fatty acid, and lactate concentrations were measured with Glucose (HK) Assay Kit (Sigma, GAHK20), Glutamine Colorimetric Assay Kit (BioVission, K556), Free Fatty Acid Quantification Colorimetric/Fluorometric Kit (BioViosn, K612) and L-Lactate Assay Kit I (Eton Bioscience, 120001), respectively.

#### Seahorse assays

Cells were seeded at 4×10^5^ cells/cm^2^ into poly-D-lysine (Sigma, P6407) coated XF96 plate (Agilent) for 4 hours prior to experiments. Complete seahorse medium was prepared from Agilent Seahorse XF Base Medium (Agilent, 102353) to contain 5.5 mM glucose, 2 mM glutamine and 1 mM pyruvate, with pH7.4. The cells were incubated in 180 μl complete seahorse medium at 37°C for 1 hour before measurements in Seahorse XFe96 Analyzer. The following working concentrations of compounds were used: 2 μM Oligomycin, 2 μM FCCP, 1 μM Rotenone, and 1 mM Antimycin A. The oxygen consumption rate (OCR) and extracellular acidification rate (ECAR) were normalized to seeded cell number. Calculation of ATP production from either glycolysis or oxidative phosphorylation is based on a published method (Mookerjee et al., 2017). See Table S1 for equations.

#### Metabolic tracing of glucose

For *in vitro* studies, cells were seeded at 1×10^5^ cells/cm^2^ into 10-cm dish for 4 hours before being switched to 10 mL cMEMα for 1 hour. The cells were then incubated for 30 mins with cMEMα containing 5.5 mM uniformly labeled ^13^C-D-glucose (^13^C_6_-Glc) (Sigma, 389374) in lieu of regular glucose. The cells were then rinsed with 10 mL ice-cold PBS and lysed with 4% perchloric acid (PCA). For *in vivo* glucose tracing, ^13^C_6_-Glc dissolved in water at 3.3 M concentration was injected at 80 mg/mouse and 60 minutes before euthanization through the tail vein of eight-week-old C57BL6/J male mice. Plasma was collected immediately before sacrifice. The tibias and femurs were immediately dissected clean of connective tissue and the bone shafts were excised free of trabecular bone with a sharp razor blade. The bone shafts were then centrifuged at 11,000 g in a table-top microcentrifuge to remove the marrow content before being homogenized in 600 ul 4% perchloric acid (PCA) in water.

Metabolite measurements were performed at the Metabolomics Core of the Children’s Hospital of Philadelphia as previously described (Nissim et al., 2012, 2014). Plasma samples and a neutralized perchloric acid (PCA) extract prepared from cell cultures or bone samples were used for measurement of ^13^C enrichment in glucose and/or TCA Cycle intermediates. Measurement was performed on either an Agilent Triple Quad 6410 mass spectrometer combined with an Agilent LC 1260 Infinity or Hewlett-Packard 5971 Mass Selective Detector (MSD), coupled with a 5890 HP-GC, GC-MS Agilent System (6890 GC-5973 MSD) or a Hewlett-Packard HP-5970 MSD using electron impact ionization with an ionizing voltage of −70eV and an electron multiplier set to 2000V. Isotopic enrichment in ^13^C aspartate isotopomers was monitored using ions at m/z[C0]418, 419, 420, 421 and 422 for M0, M1, M2, M3 and M4 (containing 1 to 4 ^13^C atoms above M0, the natural abundance), respectively. Isotopic enrichment in ^13^C lactate was monitored using ions at m/z 261, 262, 263 and 264 for M0, M1, M2 and M3 (containing 1 to 3 ^13^C atoms above natural abundance), respectively. Isotopic enrichment in ^13^C malate isotopomers was monitored using ions at m/z 419, 420, 421, 422 and 423 for M0, M1, M2, M3 and M4 (containing 1 to 4 ^13^C atoms above natural abundance), respectively. Isotopic enrichment in ^13^C fumarate isotopomers was monitored using ions at m/z 287, 288, 289, 290 and 291 for M0, M1, M2, M3 and M4 (containing 1 to 4 ^13^C atoms above natural abundance), respectively, and ^13^C enrichment in ^13^C citrate isotopomers was monitored using ions at m/z 459, 460, 461, 462, 463, 464 and 465 for M0, M1, M2, M3, M4, M5 and M6 (containing 1 to 6 ^13^C atoms above natural abundance), respectively. ^13^C enrichment in glucose was determined by LC-MS (Li et al., 2014). Organic acids levels were determined by the isotope-dilution approach and GC-MS system (Weinberg et al., 2000). ^13^C-enrichment is expressed by atom percent excess (APE), which is the fraction (%) of ^13^C enrichment above natural abundance. The level of ^13^C-labeled mass isotopomer was calculated by the product of (APE/100) times concentration and is expressed as nmoles ^13^C metabolite per gram wet weight bone, or per mg cellular protein.

#### Gene knockdown with shRNA

Lentiviral based shRNA constructs were obtained from High-Throughput Screening Core at University of Pennsylvania. Construct IDs are as follows: shEGFP, SHC005; shMe1–1, TRCN0000114877; shMe1–2, TRCN0000114878; shMe2–1, TRCN0000114867; shMe2-2, TRCN0000114870. Viruses expressing shRNA were produced by transfecting HEK293T cells with the pLKO.1 shRNA plasmid together with packaging plasmids pΔ8.2 and pVSVG by using Lipofectamine 3000 (Thermo). Cells at the various differentiation stages were dissociated as described earlier and plated at 1 × 10^5^ cells/cm^2^ for d0 cells, and 2 × 10^5^cells/cm^2^ for d4 or d7 cells, before being infected with lentiviruses at 1 transduction unit (TU)/cell for 16 hours. Infected cells were selected with 1 then 2 μg/ml puromycin for 24 hr each and then switched to cMEMα (supplemented with ascorbate and β-glycerophosphate for d4 and d7 cells) for 24 hr before the media was harvested for glucose and lactate measurements. The cells were harvested for RT-qPCR to determine the knockdown efficiency and the expression level of various genes.

#### RT-qPCR analysis

Total RNA was harvested from 2 × 10^6^ cells first lysed with 370 μL RLT buffer containing 1% 2-mercaptoethanol, and then extracted with the RNeasy mini kit (QIAGEN) according to the manufacturer’s protocol. Complementary DNA was synthesized from 1μg mRNA per reaction with the SuperScript IV VILO Master Mix with ezDNase Enzyme (Thermo). The relative expression level of specific mRNA to the 18S ribosomal RNA was determined by qPCR with PowerUp SYBR Green Master Mix (Thermo) in QuantStudio 3 Real-Time PCR System (Thermo). The relative changes were calculated with ΔΔCt method, and expressed as fold change (2^−ΔΔCt^). PCR Primer information is listed in Table S2.

#### Mitochondria copy number quantification

For total DNA extraction, 5 × 10^6^ Cell was lysed with 200 μL lysis buffer (50mM Tris, 1mM EDTA, 0.2% SDS, pH8.0) containing 0.2mg/mL Proteinase K at 55°C overnight, and then incubated with 1mg/ml RNase A at room temperature for 30–60 mins. Cell lysate was first extracted with equal volume of pH 8.0 Phenol:Chloroform:IAA with Phase Lock Gel, and then with equal volume of chloroform prior to ethanol participation with 2.5× volume of 100% EtOH and 0.1× volume of 3M pH5.2 NaOAc. DNA pellet was dissolved in TE buffer (10mM Tris, 1mM EDTA, pH8.0).

The relative abundance of the mitochondrial gene mt-Nd4 (mitochondrially encoded NADH:ubiquinone oxidoreductase core subunit 4) to the nuclear gene Hk2 (hexokinase-2) was determined by qPCR with 15 ng total DNA using SsoAdvanced Universal SYBR® Green Supermix (BioRad). The relative mitochondria copy number change was calculated with ΔΔCt method, and expressed as fold change (2^−ΔΔCt^). See Table S3 for PCR primer information.

### QUANTIFICATION AND STATISTICAL ANALYSIS

Statistical significance is calculated with two-tailed Student’s t test. All quantification graphs are presented as mean ± standard deviation. The number of biological replicates (N) is indicated in figure legends. Statistical significance is defined as p < 0.05.

## Supplementary Material

1

2

## Figures and Tables

**Table:** KEY RESOURCES TABLE

REAGENT or RESOURCE	SOURCE	IDENTIFIER
Bacterial and Virus Strains		
shEGFP (pLKO.1)	High-Throughput Screening Core at University of Pennsylvania	SHC005
shMe1-1 (pLKO.1)	As above	TRCN0000114877
shMe1-2 (pLKO.1)	As above	TRCN0000114878
shMe2-1 (pLKO.1)	As above	TRCN0000114867
shMe2-2 (pLKO.1)	As above	TRCN0000114870
Chemicals, Peptides, and Recombinant Proteins		
(+)-Etomoxir sodium salt hydrate	Sigma	E1905
2-Deoxy-D-glucose	Sigma	D6134
2-Mercaptoethanol	BioRad	1610710
Antimycin A	Sigma	A8674
Ascorbic acid	Sigma	A4544
Ascorbic acid-free MEMa	Thermo	A10490
Bovine Serum Albumin	Proliant Health&Biologicals	7500804
BPTES	Sigma	SML0601
Carbonyl cyanide 4-(trifluoromethoxy) phenylhydrazone	Sigma	C2920
Collagenase I	Sigma	C0130
Customized ascorbic acid-free MEMa	GIBCO	ME18459P1
D-(+)-Glucose	Sigma	G7021
D-(+)-Galactose	Sigma	G5388
DPBS	Thermo	14190250
FBS	Thermo	26140087
HBSS	Thermo	14025
L-Glutamine	Thermo	25030081
O-(Carboxymethyl)hydroxylamine hemihydrochloride	Sigma	C13408
Oligomycin	Sigma	O4876
Penicillin-Streptomycin	Thermo	15140122
5 PRIME Phase Lock Gel Light	Fisher	FP2302800
poly-D-lysine	Sigma	P6407
propidium iodide	Thermo	P1304MP
Proteinase K	Invitrogen	25530–015
RNase A	Sigma	10109142001
Rotenone	Sigma	R8875
Sodium acetate	Sigma	S2889
Sodium Oleate	Sigma	O3880
Sodium oxamate	Sigma	O2751
Sodium Palmitate	Sigma	P9767
Sodium pyruvate	Sigma	P5280
UK-5099	Sigma	PZ0160
UltraPure Phenol:Chloroform:Isoamyl Alcohol (25:24:1, v/v)	Thermo	15593049
Uniformly labeled ^13^C-D-glucose	Sigma	389374
β-glycerol phosphate	Sigma	G9422
Critical Commercial Assays		
CellTiter-Glo® Luminescent Cell Viability Assay	Promega	G7570
Free Fatty Acid Quantification Colorimetric/ Fluorometric Kit	BioViosn	K612
Glucose (HK) Assay Kit	Sigma	GAHK20
Glutamine Colorimetric Assay Kit	BioViosn	K556
L-Lactate Assay Kit I	Eton Bioscience	120001
PowerUp SYBR Green Master Mix	Thermo	A25778
RNeasy Mini Kit	QIAGEN	74106
SsoAdvanced Universal SYBR® Green Supermix	BioRad	1725275
SuperScript IV VILO Master Mix with ezDNase Enzyme	Thermo	11766500
Deposited Data		
Raw and analyzed data	This paper	GEO: GSE154991
Experimental Models: Organisms/Strains		
Mouse: C57BL/6J	The Jackson Laboratory	000664
Mouse: Tg(Col1a1*2.3-GFP)1Rowe/J	The Jackson Laboratory	013134
Oligonucleotides		
Primers for RT-qPCR, see Table S2	This paper	N/A
Primers for DNA-qPCR, see Table S3	This paper	N/A

## Highlights

- Glycolysis produces 80% of the energy in osteoblasts under aerobic conditions
- Lactate is the predominant metabolic fate for glucose in bone *in vivo*
- Mitochondrial respiration is diminished during osteoblast maturation
- Me2 funnels glucose carbons into malate-aspartate shuttle to sustain glycolysis
