# Supplementary material for: Malic Enzyme Couples Mitochondria with Aerobic Glycolysis in Osteoblasts
Source: Cell Rep. Author manuscript; Available in PMC 2021 Jun 7. (PMC8183612; doi:10.1016/j.celrep.2020.108108)
Supplement: 1 [file NIHMS1627869-supplement-1.pdf]

Cell Reports, Volume *32*

Supplemental Information

Malic Enzyme Couples Mitochondria with Aerobic Glycolysis in Osteoblasts

Wen-Chih Lee, Xing Ji, Itzhak Nissim, and Fanxin Long

Supplemental Materials

Supplemental Fig. S1

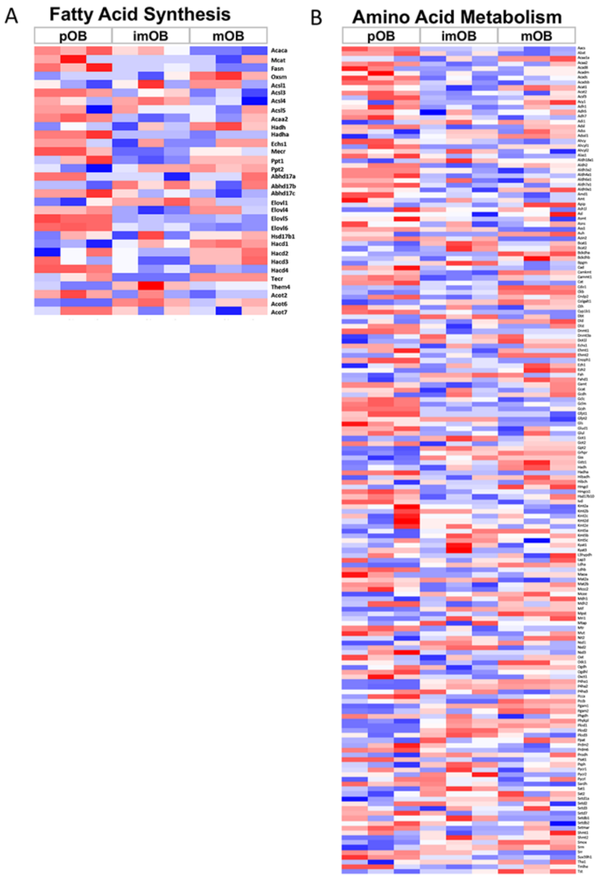

Figure S1. Heatmaps of gene expression levels by RNA-seq. Related to Figure 6. (A) Genes involved in amino acid metabolism. (B) Genes involved in fatty acid synthesis. pOB: preosteoblasts at d0; imOB: immature osteoblasts at d4 of differentiation; mOB: mature osteoblasts at d7 of differentiation. N=3 for each stage.

Supplemental Fig. S2

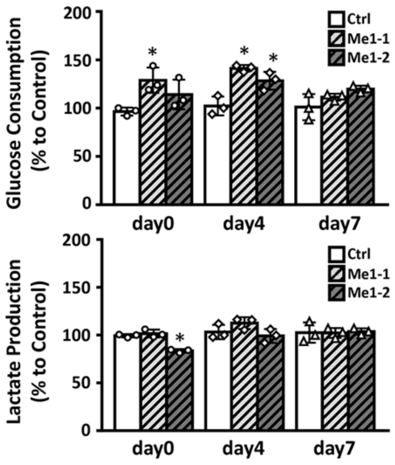

Figure S2. Effects of Me1 knockdown on glucose consumption or lactate production. Related to Figure 7. Ctrl: shRNA against EGFP; Me1-1: shRNA #1 against Me1; Me1-2: shRNA #2 against Me1. *: $p<0.05$, n=3. Error bars: std.

Table S1. Equations for calculations of ATP production from Seahorse data. Related to STAR Methods section.

Calculated Values	Equations
OCR_{mito}	$= (OCR_{total} - OCR_{rot/myc})$
$OCR_{coupled}$	$= (OCR_{total} - OCR_{oligo})$ * hyperpolarization correction factor
PPR_{total}	$= (ECR_{total}$ / buffering power)
PPR_{resp}	$= (OCR_{mito} * (\text{max } H^+/O_2) * ((10^{pH-pK1}) / (1 + 10^{pH-pK1}))) = (OCR_{mito} * K_{PPRresp}))$
PPR_{glyc}	$= (PPR_{total} - PPR_{resp})$
Glycolytic ATP ($J_{ATPglyc}$)	$= (PPR_{glyc}$ * ATP/lactate) + $(OCR_{mito} * 2 * ATP/[O]_{glyc})$
Oxidative ATP (J_{ATPox})	$= (OCR_{coupled} * 2* ATP/[O]_{oxphos}) + (OCR_{mito} * 2 * ATP/[O]_{TCA})$

Parameters	Values	Parameters	Values
buffering power (mpH/pmol H^+)	0.045	$K_{PPRresp}$	0.953
pK_1 at 37°C (CO_2 -> HCO_3^-)	6.093	$ATP/[O]_{glyc}$	0.167
hyperpolarization correction factor	0.908	$ATP/[O]_{oxphos}$	2.486
max H^+/O_2	1.000	$ATP/[O]_{TCA}$	0.121
ATP/lactate	1.000		

Table S2. Primers for RT-qPCR. Related to STAR Methods section.

Primer	Sequence
Alpl_Mm_F	ACTGATGTGGAATACGAACTGG
Alpl_Mm_R	AGTTCAGTGCGGTTCCAG
Atf4_Mm_F	ATGGCGTATTAGAGGCAGC
Atf4_Mm_R	CTTTGTCCGTTACAGCAACAC
Bglap2_Mm_F*	CACCTAGCAGACACCATGAG
Bglap2_Mm_R*	GTTCACTACCTTATTGCCCTCC
Col1a1_Mm_F	AGCCGCAAAGAGTCTACATG
Col1a1_Mm_R	CTTAGGCCATTGTGTATGCAG
Ldha_Mm_F	GCTCCCCAGAACAAGATTACAG
Ldha_Mm_R	TCGCCCTTGAGTTTGTCTTC
Me1_Mm_F	AGTATCCATGACAAAGGGCAC
Me1_Mm_R	ATCCCATTACAGCCAAGGTC
Me2_Mm_F	TGTTATTCTCTGTGAAGCCCG
Me2_Mm_R	TGAATATTAGCCAGCGACGG
Rn18s_Mm_F	CGGCTACCACATCCAAGGAA
Rn18s_Mm_R	GCTGGAATTACCGCGGCT
Sp7_Mm_F	ATCTTCCACTTCGCCTGC
Sp7_Mm_R	AACCAATGGGTCCACCGC

* By Primer-BLAST from NCBI, this primer set also recognize Bglap and Bglap3.

Table S3. Primers for DNA-qPCR. Related to STAR Methods section.

Primer	Sequence
Hk2_Mm_gDNA_F	GCCAGCCTCTCCTGATTTTAGTGT
Hk2_Mm_gDNA_R	GGGAACACAAAAGACCTCTTCTGG
mtNd4_Mm_gDNA_1F	CATCACTCCTATTCTGCCTAGC
mtNd4_Mm_gDNA_1R	GTGGCTATAAGTGGGAAGACC
mtNd4_Mm_gDNA_2F	AGCTCAATCTGCTTACGCCA
mtNd4_Mm_gDNA_2R	TGTGAGGCCATGTGCGATTA
